# MRI assessment of autologous osteochondral transplantation in talus: correlation with clinical outcomes and second look

**DOI:** 10.3389/fspor.2025.1657265

**Published:** 2026-01-29

**Authors:** Sanbiao Liu, Yunfeng Chu, Wen Zhou, Yuxin Yan, Yuyi Zhang, Sumeng Chen, Lu Bai

**Affiliations:** 1 Department of Hand and Microvascular Surgery, Shenzhen Hospital, Peking University, Shenzhen, Guangdong Province, China; 2 Department of Medical Imaging, Shenzhen Hospital, Peking University, Shenzhen, Guangdong Province, China; 3 Department of Sports Medicine, Shenzhen Hospital, Peking University, Shenzhen, Guangdong Province, China

**Keywords:** osteochondral lesions, talus, autologous osteochondral transplantation, magnetic resonance, second-look arthroscopy

## Abstract

**Background:**

Autologous osteochondral transplantation (AOT) is an effective technique for treating complex osteochondral injuries of the talus. However, there is still controversy regarding the imaging assessment of its postoperative efficacy. MRI, as a non-invasive examination, is the primary method for evaluating surgical outcomes, while invasive secondary arthroscopic surgery provides a more direct and accurate evaluation of intra-articular results. The correlation between these two assessment methods and clinical outcomes remains unclear.

**Purpose:**

To evaluate the correlation between MRI findings assessed using the MOCART scoring system and functional outcomes, as well as arthroscopic second look, in patients undergoing (AOT) for osteochondral lesions of the talus.

**Methods:**

A retrospective analysis was conducted on 47 patients. All patients were followed for a minimum of two years postoperatively. Functional evaluations were performed at one and two years after surgery using the American Orthopaedic Foot and Ankle Society (AOFAS) score and the Visual Analog Scale (VAS). Imaging assessments utilized the Magnetic Resonance Observation of Cartilage Repair Tissue (MOCART) MRI scoring system. All patients underwent secondary arthroscopy for internal fixation removal at the two-year mark, during which the International Cartilage Repair Society (ICRS) scores were recorded.

**Results:**

There was a low correlation between the MOCART scores and both functional scores and arthroscopic scores postoperatively. At one year post-surgery, the MOCART score showed a low correlation with the AOFAS score (*r* = 0.27, *p* = 0.07, 95% CI: −0.01–0.36). By two years post-surgery, the MOCART score demonstrated a low correlation with the AOFAS score (*r* = 0.34, *p* = 0.02, 95% CI: 0.05–0.49), VAS score (*r* = −0.46, *p* < 0.05, 95% CI: −0.08 to −0.02), and ICRS score (*r* = 0.36, *p* < 0.05, 95% CI: 0.40–3.11). ICRS and AOFAS scores (*r* = 0.56, *p* < 0.05, 95% CI: 1.19–3.07), indicating a moderate correlation.

**Conclusion:**

In autologous osteochondral transplantation (AOT) for the talus, the MOCART scores showed a low correlation with clinical function or secondary arthroscopic scores. The MRI assessment of talar cartilage repair requires more detailed evaluation.

**Level of evidence:**

4.

## Introduction

Osteochondral lesions of the talus (OLTs) are a prevalent clinical condition that cause pain and dysfunction in the ankle, impairing quality of life and posing a substantial burden on those affected (1, 2). There are various treatment options for osteochondral lesions of the talus (OLTs) depending on the condition. For small lesions, conservative treatment may be effective. Bone marrow stimulation is recommended when the lesion area is less than 1.5 cm^2^ (3–5). However, for lesions larger than 10 mm in diameter accompanied by subchondral cyst formation, autologous osteochondral transplantation (AOT) is recommended (6–9) its effectiveness and practicality have been clinically recognized (10–12).

The MRI-based Magnetic Resonance Observation of Cartilage Repair Tissue (MOCART) score is a widely utilized tool for assessing cartilage repair following AOT (13, 14). However, several studies have reported a limited correlation between postoperative MOCART scores and functional outcomes, suggesting that the MOCART score may not fully reflect the clinical benefits of the surgical intervention (15–17). Additionally, the International Cartilage Repair Society (ICRS) score, based on second-look arthroscopy, is regarded as the gold standard for assessing cartilage repair, as it enables direct visualization and a more comprehensive evaluation of the repaired cartilage (18–20). This scoring system classifies cartilage damage based on characteristics such as surface integrity, depth, and tissue morphology observed during direct arthroscopic visualization. It is commonly used to grade cartilage damage in arthroscopic procedures (21). The primary objective of this study was to assess the correlation between second-look arthroscopy (ICRS), functional scales, and the MOCART score.

## Methods

### Patient selection

**Figure 1:**
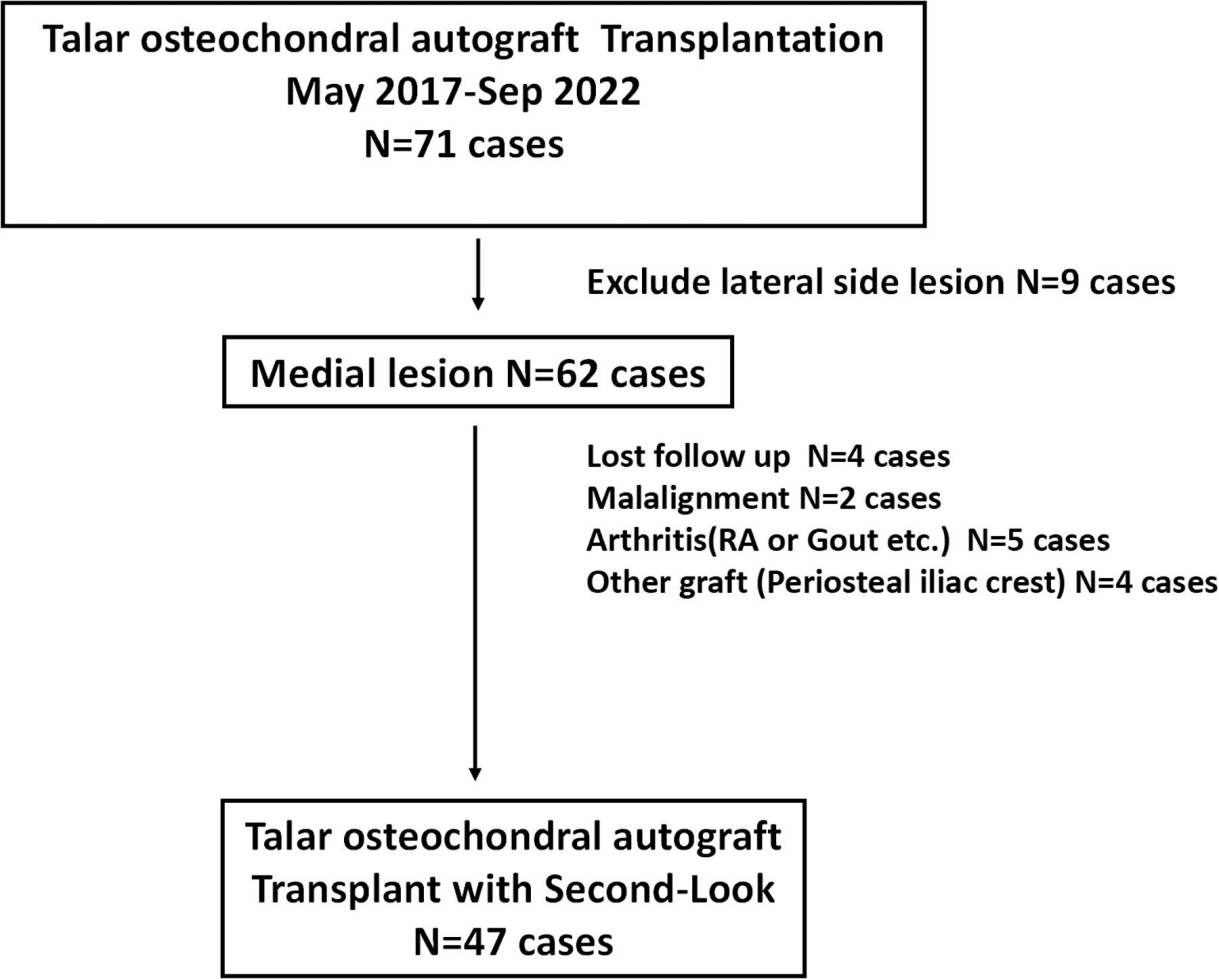
The study flowchart summarizing screening, inclusion and exclusion of the patients.

The study protocol was established according to the ethical guidelines of the Helsinki Declaration and was approved by the Human Ethics Committee of Hospital. A retrospective analysis of patients with OLT treated in our hospital from May 2017 to September 2022 was conducted. Inclusion criteria were: (1) previous conservative treatments [e.g., rest, bracing, nonsteroidal anti-inflammatory drugs (NSAIDs)] administered for >3 months were ineffective; (2) OLT with a subchondral cyst >10 mm in diameter; (3) regular follow-up and second-look arthroscopy 2 years after surgery; Exclusion criteria were: (1) ankle or hindfoot malalignment; (2) history of systemic diseases with intra-articular infiltration (e.g., intra-articular gout crystals, rheumatoid arthritis); (4) Use Iliac bone graft; (5) The lesion is located on the lateral side of the talus. Patient selection is shown in Figure 1.

### Surgical procedure and rehabilitation

The procedure was performed under spinal anesthesia with the patient in the supine position. Standard arthroscopic exploration was conducted to debride osteophytes and synovitis tissue, followed by medial malleolar osteotomy for optimal exposure and thorough debridement of the osteochondral lesion. An appropriately sized osteochondral graft was harvested and securely implanted into the defect area and match the curvature of the surrounding cartilage. The osteotomy was stabilized with 2–3 cannulated screws.

Postoperatively, the affected limb was immobilized in a short-leg cast for two weeks, then followed by an ankle joint activity brace. Six weeks after surgery, partial weight-bearing was initiated, progressing to full weight-bearing as tolerated.

### Follow-up assessments

The follow up data were recorded 1 and 2 years after surgery. Functional scale [American Orthopedic Foot and Ankle Society (AOFAS) Ankle-Hindfoot Scale] and Visual Analog Scale (VAS) were evaluated.

Two years after surgery, patients underwent second-look arthroscopy during hardware removal. At that time, the International Cartilage Repair Society (ICRS) scores of the repaired cartilage were recorded. Second-look arthroscopy records were randomized, and assessments were performed by two independent observers to minimize bias.

### Radiological evaluation

The MR examination was performed using a 3.0T MRI scanner (Siemens Spectra 3.0T) with a dedicated 16-channel ankle coil. The patient was positioned supine with the foot placed in the coil in a neutral position, aligning the coil center with the Achilles tendon region. All sequences were acquired with the following parameters: slice thickness 3 mm, interslice gap 0.3 mm. Axial and sagittal images were obtained with 3 mm section thickness and 0.3 mm gap. The MRI protocols included: T1-weighted imaging (T1-WI), PD/T2-weighted fast spin-echo (T2-WI, PD-WI), and two-dimensional FLASH-weighted spin-echo (GRE-WI). The MOCART score were used to assess the repaired cartilage. The intraclass correlation coefficient (ICC) was calculated to assess inter-observer reliability of MOCART score, demonstrating satisfactory agreement (ICC = 0.92, 95%CI 0.88–0.95).

### Statistical analysis

Statistical analysis was performed using IBM SPSS 24 for Windows (SPSS, Inc., Chicago, IL, USA). *A priori* power analysis (G*Power 3.1; *α* = 0.05, *β* = 0.2) determined that 28 cases would provide 80% power to detect clinically relevant correlations (*ρ* ≥ 0.5) based on prior OLT studies (8, 13), though this threshold precluded more complex analyses. Data normality was assessed with the Kolmogorov–Smirnov test. Preoperative and postoperative outcomes were compared using the paired *t*-test or the Wilcoxon signed-rank test. Spearman's rank correlation and a 95% confidence interval (CI), computed via Fisher's z-transformation, were used to evaluate the correlation between variables. Correlation strength was interpreted as follows: 0.00–0.30 (negligible), 0.30–0.50 (low), 0.50–0.70 (moderate), 0.70–0.90 (high), and 0.90–1.00 (very high). A *P*-value of <0.05 was considered statistically significant. Calculate the sample size using PASS 12.0 software. Choose a correlation confidence level of 90% and a confidence interval of 95%. The minimum sample size were 43 cases.

## Results

**Figure 2:**
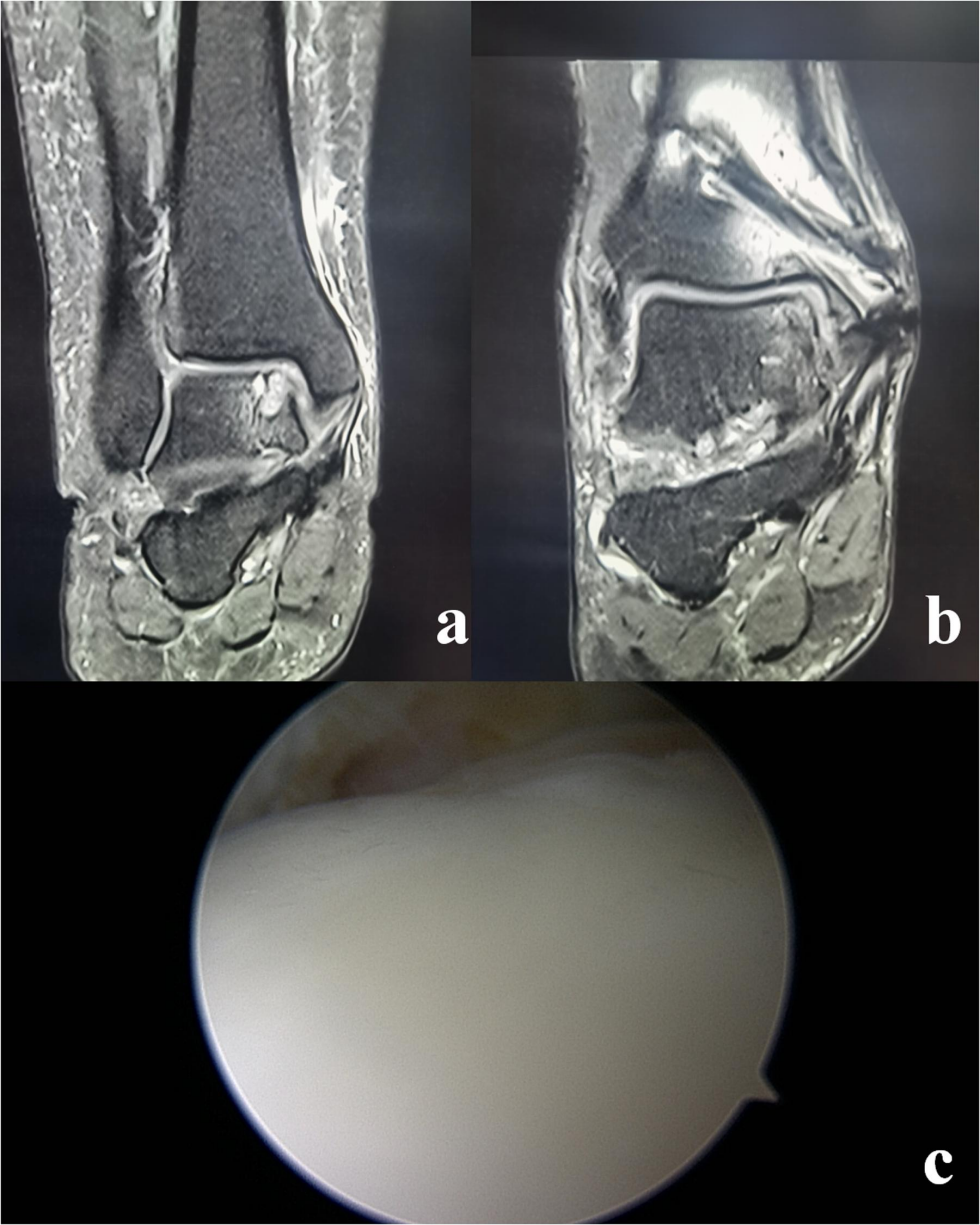
Typical and representative findings in a patient. **(a)** Magnetic resonance imaging (MRI) showed a large cartilage injury with a subchondral cyst on the medial zone of talus. **(b)** At the 2-year follow-up, MRI showed a healed graft with smooth cartilage surface. **(c)** Second-look arthroscopic examination showed nearly normal cartilage.

This study included 47 patients (35 male and 12 female), with a mean age of 35.1 years (range 26–52 years). The mean follow-up duration was 32.5 ± 6.7 months (range 24–51 months), and the average body mass index (BMI) was 24.3 kg/m^2^ (Table 1). All patients reported a history of previous ankle sprains on the affected side, 8 patients were diagnosed with anterior talofibular ligament injury through physical examination and MRI examination. Preoperative magnetic resonance imaging (MRI) measurements revealed that the mean horizontal diameter of the lesion on talus surface was 10.5 ± 0.7 mm, the mean AP diameter was 11.9 ± 1.1 mm, and the mean depth of the lesion was 10.0 ± 1.0 mm. Typical patient shows in Figure 2.

**Table 1:** Baseline characteristics.

Variable	Total
Number of patients	47
Age, years	35.1 ± 5.4
Follow-up, months	32.5 ± 6.7
BMI (kg/m^2^)	24.3 ± 3.1
AOFAS	73.1 ± 4.1
VAS	4.0 ± 0.9
ICRS	4.0 ± 1.0
lesion size(mm)	
Length	11.9 ± 1.1
Width	10.5 ± 0.7
Depth	10.0 ± 1.0

**Figure 3:**
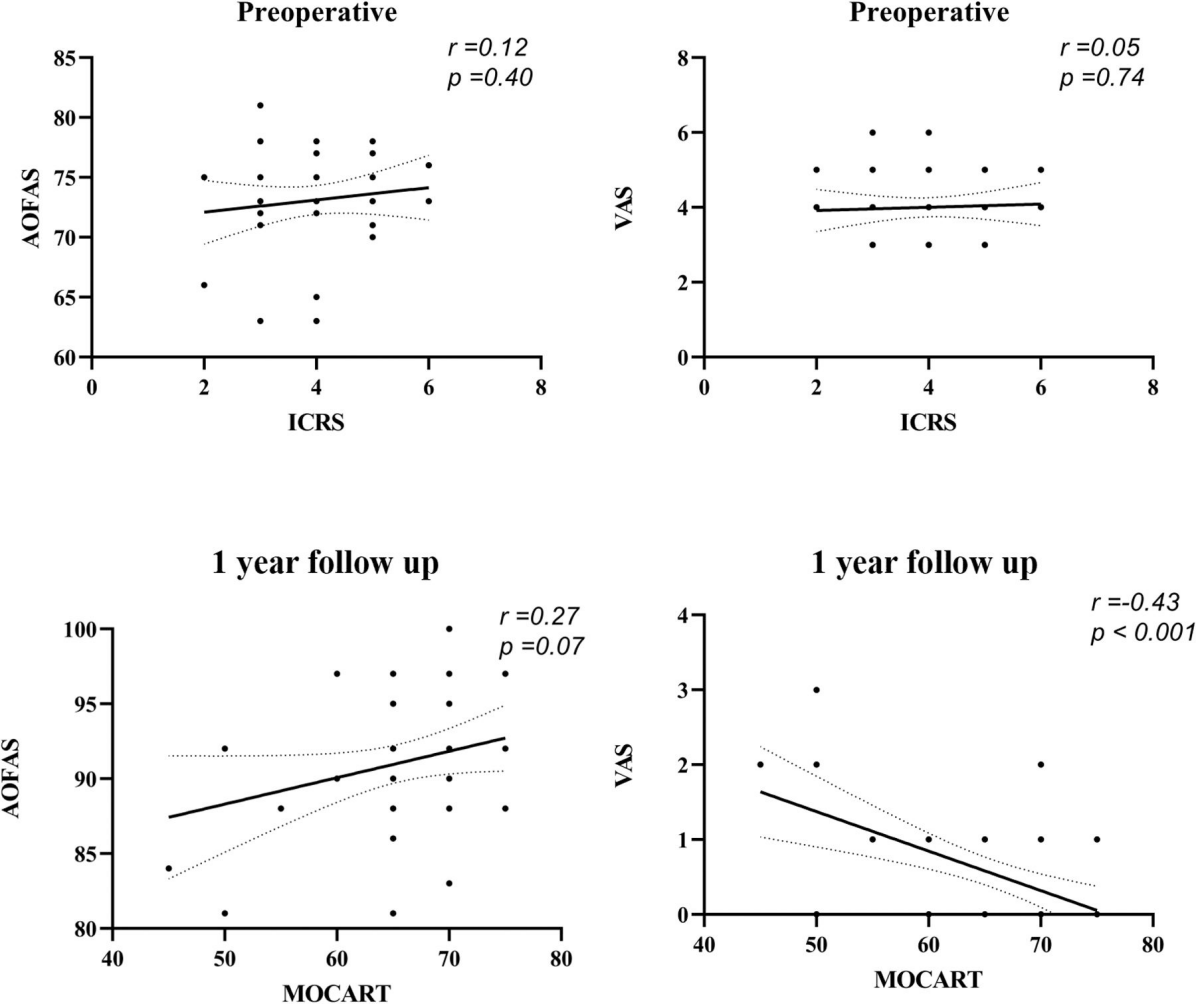
Correlation between functional, radiological, and second-look arthroscopy results at preoperative and 1 year after autologous osteochondral transplantation.

**Figure 4:**
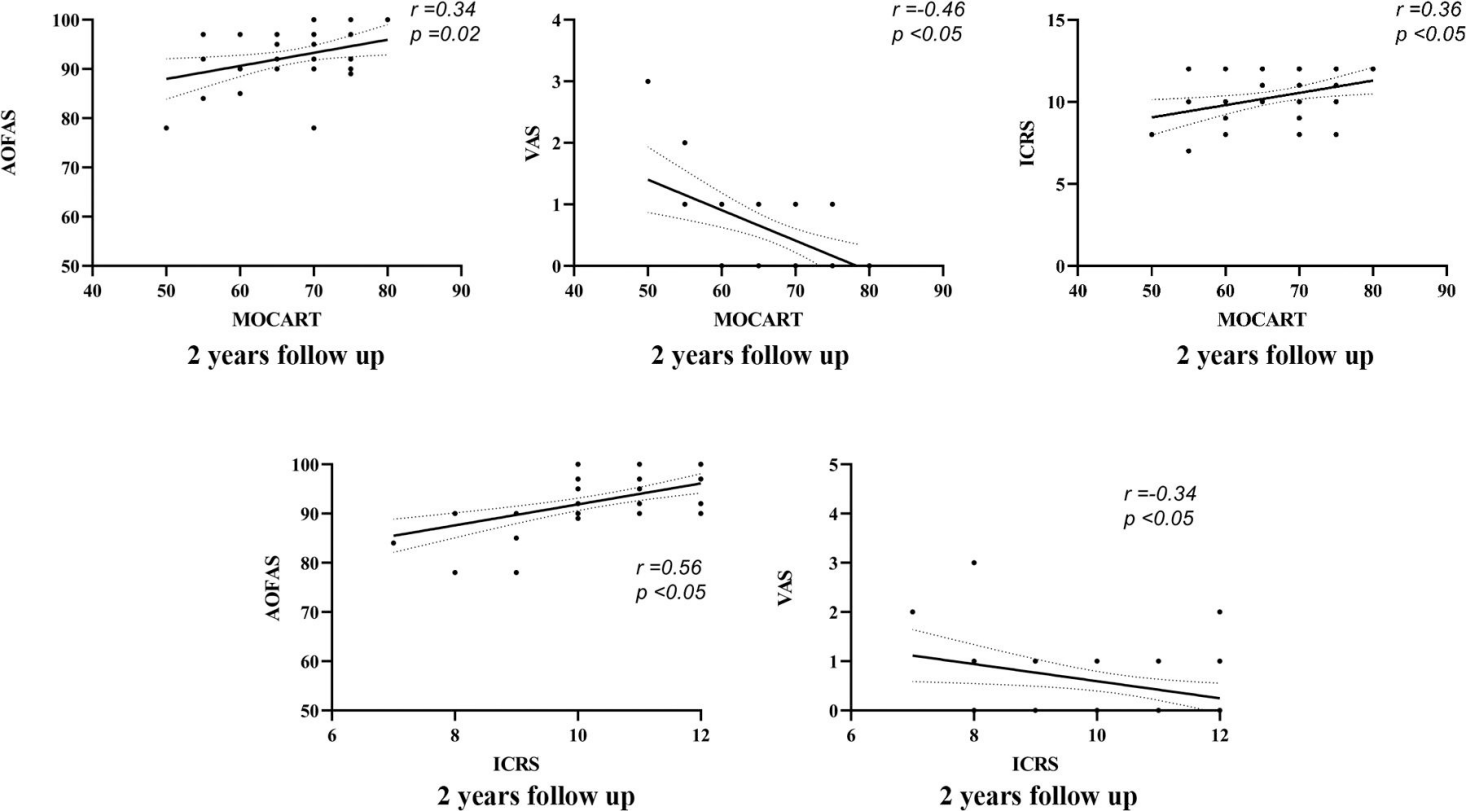
Correlation between functional, radiological, and second-look arthroscopy results at preoperative and 2 year after autologous osteochondral transplantation.

AOFAS, VAS, ICRS, and MOCART scores showed improvement during postoperative follow-up, the scores are shown in Table 2. Pearson correlation tests were used to assess the relationships between AOFAS, VAS, MOCART, and ICRS scores at various follow-up periods. The results are illustrated in Figures 3, 4, showing the correlations between MOCART scores and AOFAS, VAS, and ICRS scores at different time points. The initial ICRS score showed a negligible correlation with preoperative AOFAS (*r* = 0.12, *p* = 0.40, 95% CI: −0.69–1.71) and VAS scores (*r* = 0.05, *p* = 0.74, 95% CI: −0.21–0.30), with *r* < 0.30.

**Table 2:** Preoperative and postoperative data: function data, radiographic and arthroscopy results.

Function data, score	Preoperative Mean ± SD (Range)	Postoperative 1 year Mean ± SD (Range)	Postoperative 2 years Mean ± SD (Range)	*P_1_* Value	*P_2_* Value
AOFAS	72.9 ± 4.1 (63 to 81)	91.1 ± 4.3 (81 to 100)	92.9 ± 4.7 (78 to 100)	<.001	<.001
VAS	4.0 ± 0.9 (3 to 6)	0.6 ± 0.7 (0 to 3)	0.5 ± 0.7 (0 to 3)	<.001	0.323
Radiographic results, score					
MOCART		65.1 ± 6.5 (45 to 75)	67.1 ± 6.6 (50 to 80)		<.001
Arthroscopy results, score					
ICRS	4.1 ± 1.1 (2 to 6)	10.2 ± 1.3 (8 to 12)		<.001	

Abbreviations: AOFAS, American Orthopaedic Foot & Ankle Society; VAS, visual analog scale; ICRS, The International Cartilage Repair Society; MOCART, magnetic resonance observation of cartilage repair tissue.

*P*_1_ value: comparison of results before preoperative and 2 years postoperatively.

*P_2_* value: comparison of results before 1 year postoperatively and 2 years postoperatively.

**Figure 5:**
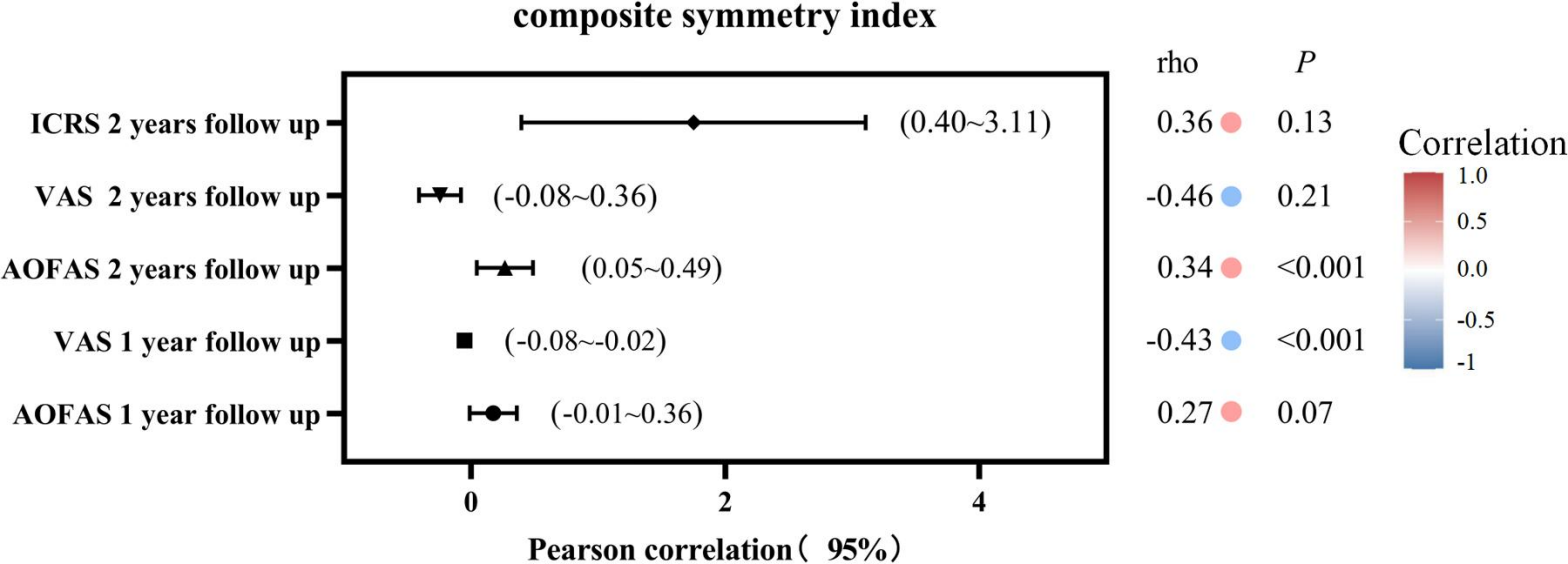
Plot showing the 95% confidence intervals for the Pearson correlations between ICRS Score,clinical scores and MOCART Score.

At 1 year postoperatively, a weak correlation was observed between MOCART and VAS scores (*r* = −0.43, *p* < 0.001, 95% CI: −0.08 to −0.02). A similarly weak correlation was found between MOCART and AOFAS scores (*r* = 0.27, *p* = 0.07, 95% CI: −0.01–0.36). At 2 years postoperatively, the highest correlation was observed between ICRS and AOFAS scores (*r* = 0.56, *p* < 0.05, 95% CI: 1.19–3.07), indicating a moderate correlation. In contrast, correlations between MOCART and AOFAS (*r* = 0.34, *p* = 0.02, 95% CI: 0.05–0.49), VAS (*r* = −0.46, *p* < 0.05, 95% CI: −0.08 to −0.02), and ICRS (*r* = 0.36, *p* < 0.05, 95% CI: 0.40–3.11) remained weak (Figure 5).

## Discussion

Our retrospective review showed that transplanted hyaline cartilage survived, and ICRS scores improved in all patients at the 2-year follow-up after AOT. A weak correlation was observed between MOCART and function scores at 2 years postoperatively, and low to moderate correlation between ICRS scores and functional scores.

MRI is currently the most commonly used imaging method for observing cartilage condition (22, 23). However, among the follow-up indicators after AOT, MRI often reveals “poor signals,” such as bone marrow edema around the graft, signal mismatch between the graft and surrounding tissue, and the formation of subchondral cysts (22, 24). Previous studies have shown no significant correlation between MRI performance at an early follow-up and clinical functional results (22, 24, 25). Our results are in agreement with these studies and also found weak correlation between them. The incidence of cyst formation after AOT is reportedly 65% (16), and the incidence of cyst formation after AOT concurrent with extracellular matrix enhancement is 14% (10). Cyst formation could be attributed to the joint synovial fluid flowing into the subchondral bone through the graft–host cartilage connection interface after AOT. The signal of subchondral bone edema is universal. According to Dhollander et al. (26), all cases exhibited high levels of subchondral edema following knee osteochondral transplantation. Pain in patients with OLT was attributed to subchondral synovial fluid entering the subchondral bone through microfractures, stimulating nerve endings and causing pain (27). After AOT, even if the graft appears to fit well with the surrounding tissue, synovial fluid can still enter the subchondral bone through the surgical gap, leading to postoperative bone marrow edema and the formation of subchondral cysts. Over time, the graft heals with the surrounding subchondral bone, preventing further synovial fluid infiltration. However, this does not cause significant discomfort, which may explain the lack of a clear correlation between functional and radiological scores in the long term post-surgery.

Similarly, previous studies have suggested that the correlation between the results of second-look arthroscopy and functional results is limited (28–30). In our study, at 2 years postoperatively, AOFAS and ICRS scores showed a moderate correlation, while no significant correlation was found between the VAS and ICRS scores, which aligns with current knowledge on this topic. Second-look arthroscopy can truly reflect the condition of the articular surface. For patients with good graft healing, the condition of the subchondral bone or cyst cannot be observed. Furthermore, there are many causes of postoperative pain, including local synovitis, scar adhesion, or psychological factors (27). Therefore, it is reasonable to believe that when the surface of the articular cartilage is intact, second-look ICRS score would be good with or without pain.

For MOCART and ICRS scores, there is a certain similarity between the evaluation of the consistency of the graft, surrounding cartilage and the articular surface. Goller et al. reported MOCART 2.0 parameters showed significant correlation with Delta International Knee Documentation Committee Subjective Form (IKDC) scores in the postoperative course after retropatellar matrix-associated chondrocyte transplantation (MACT) (31). This research differ with our finding. However, the talus's convex surface and load-bearing mechanics (vs. retropatellar cartilage in Goller's study) may reduce MOCART's sensitivity to functional outcomes. This may indicate that the matrix-associated chondrocyte technique was more suitable for MOCART scale instead of AOT procedure. Furthermore, the ICRS score may not change even if MRI finds abnormal subchondral bone signals or new cysts. This may be a limitation of the ICRS score because observing only the surface of the graft will indeed lead to some pathological changes being missed, even though these conditions may be routine manifestations after AOT. In our study, there was no significant correlation between ICRS and MOCART scores. Although the ICRS score is the gold standard for cartilage evaluation, it is occasionally difficult to judge the condition of the subchondral bone, even if the changes in subchondral bone may be normal. Therefore, it may be more reasonable to combine the MOCART score with the ICRS score for an integrated evaluation of the graft.

This study also has limitations. First, More functional scores can be included in the follow-up process to comprehensively analyze correlations. Previous study reported that the Foot and Ankle Outcome Score (FAOS) as patient-specific outcome measures for the surgical treatment of OLT (29). Second, we did not collect cartilage specimens for histological analysis. Finally, since the internal fixators were taken out after 2 years from the operation, the follow-up time was set to 2 years, and we did not assess the patients for new positive findings in the long-term follow-up after removing the internal fixators. In the future, longer-term functional and imaging follow-up studies are needed to verify the current conclusions. Fourth, the relatively small sample size constitutes another limitation. Although our power analysis confirmed the adequacy of the current sample size for correlation analyses (*β* = 0.8, *α* = 0.05), it remains insufficient for more complex multivariate regression modeling. This sample size restriction. Fifth, future studies should incorporate advanced cartilage-specific MRI sequences (e.g., T2 mapping, T1*ρ*) for more sensitive evaluation of graft integrity.

In conclusion, AOT is an effective treatment for Hepple V OLT. However, the correlation between second-look arthroscopy findings and functional scores was weak, and a low correlation was found between arthroscopy results and MRI findings after AOT.

## Data Availability

The original contributions presented in the study are included in the article/Supplementary Material, further inquiries can be directed to the corresponding author.
